# Exploring the Link Between Visual Attention to Familiar or Novel Food Stimuli and Food Choice Using Integrated Electroencephalography and Eye Tracking: Protocol for Nonrandomized Pilot Study

**DOI:** 10.2196/69541

**Published:** 2025-05-21

**Authors:** Farshad Arsalandeh, Ali Shahbazi, Mohammad Ali Nazari

**Affiliations:** 1 Department of Neuroscience Faculty of Advanced Technologies in Medicine Iran University of Medical Sciences Tehran Iran

**Keywords:** attentional bias, eye tracking, stimuli familiarity, dot-probe, electroencephalography

## Abstract

**Background:**

Understanding the factors influencing food choice is critical for developing effective strategies to promote healthier eating habits and creating policies that support public health. Attentional bias, the inclination to focus attention on specific stimuli, plays a significant role in shaping food preferences by affecting how individuals perceive and react to various food-related elements. Various methodologies exist to examine attentional bias, including the dot-probe task, which measures reaction times to probes appearing after paired stimuli (eg, novel vs familiar food images); eye-tracking, which tracks gaze patterns and fixations to determine visual attention; and electroencephalography, which records brain activity, capturing early and late neural responses (eg, N100, P300) linked to attention processing; however, integrated approaches combining these methods to assess bias toward familiar versus novel foods remain underexplored.

**Objective:**

This study aims to examine differences in attention toward familiar versus novel food stimuli using integrated eye-tracking, dot-probe, and electroencephalography methods, and to explore associations with self-reported food choice.

**Methods:**

A total of 40 healthy adult participants will be recruited. Participants will be presented with pairs of familiar or novel food images, while their visual attention and brain activity are recorded concurrently. Eye-tracking metrics, including time to first fixation and total fixation duration, will be used to assess visual attention. Electroencephalography data will be collected to measure the amplitude of event-related potential components, such as P300 and N100, associated with attentional processing. Reaction times will also be recorded as a behavioral measure of attentional engagement with familiar versus novel food items. Data analysis will involve repeated measures ANOVA to examine the effects of food familiarity and novelty on attentional bias metrics. Correlation analyses will also be conducted to explore the relationships between eye-tracking, electroencephalography, and dot-probe measures.

**Results:**

This study was approved by the Ethics Committee of the Iran University of Medical Sciences in February 2021 and funded in January 2022. Data collection began in November 2022 and is expected to be completed in July 2025. As of the submission of this study, 36 individuals have been recruited. Data analysis has not yet commenced, but it is planned to begin upon the completion of data collection. The results are anticipated to be published by December 2025. The protocol was registered with the Open Science Framework in September 2024.

**Conclusions:**

The main outcome of this study is identifying differences in attentional bias metrics toward familiar versus novel food stimuli at different presentation times. These findings will provide preliminary data on the application of an integrated approach for capturing attentional bias to food-based stimuli based on their familiarity or novelty, and how these biases may be linked to food choice behaviors.

**International Registered Report Identifier (IRRID):**

DERR1-10.2196/69541

## Introduction

Food decision, including choices around types, timing, and portions, is a response shaped by various stimuli and directly influences both energy and nutrient intake [1,2]. Food choices are influenced by individual preferences and postconsumption reactions to food intake. The attention individuals give to food depends on the stimuli that automatically act on them (bottom-up factors) and is also determined by their interests and goals (top-down factors) [3,4]. Bottom-up and top-down processing occur simultaneously and interact with each other [5]. Bottom-up factors act automatically on the consumer and include certain characteristics for processing basic stimuli such as size, color, or shape [6]. Top-down factors are related to the person themselves [7]. They include the consumer’s voluntary search for and attention to specific food product information [8], as well as the processing of individual experiences, motivations, and expectations. In research on the impact of information provision on food choices, it is often assumed that individuals attend to all provided information. However, individuals may not notice or perceive all the information presented [9]. If information is unattended to, it cannot inform choice [10]. Thus, it is important to evaluate if and how people pay attention to information, and the factors influencing such attention or inattention.

Visual attention is an important factor in the food decision-making process, providing a key contribution to subsequent decision-making [7]. Understanding attentional bias, where individuals preferentially process specific types of information, has been critical in understanding food-related behaviors in the context of consumer behavior or etiology of diseases, namely obesity [11]. Attentional bias has been measured using novel psychophysiological methods, including eye-tracking [12]. Eye-tracking technology provides an objective way to measure eye fixation duration and movement, capturing visual attention toward prominent stimuli [13,14]. In eye-tracking, participants are presented with pictures, either concurrently with reaction- and time-based paradigms or with instructions to simply gaze at pictures presented. One well-documented reaction time paradigm is the dot-probe task. In this task, participants are presented with cues of varying duration, which impacts the interpretation of attentional bias [15]. Previous research suggested stimulus presentation lengths of ≤200 milliseconds being associated with automatic orientation of attention, whereas presentation lengths ≥500 milliseconds are associated with maintained attention [16]. Currently, there are several studies in the literature applying either eye tracking or dot-probe to investigate the attentional bias to different food or nonfood stimuli among the healthy population or across disorders, such as anxiety, obesity, and eating disorders [17-20]. While visual probe tasks are the most commonly implemented task assessing food-related attentional bias, some current evidence has called their test-retest reliability into question and suggests combining different methods of attentional bias assessment for obtaining more reliable results [21].

While some previous studies have suggested a role for visual attentional bias toward familiarity and novelty of stimuli [22], there is a paucity of studies using electroencephalography to examine this, particularly in the context of food choice behavior. Electroencephalography is a noninvasive technique used to measure brain activity in response to stimuli by recording event-related potentials (ERPs). So far, only a few studies have explored integrating electroencephalography, eye-tracking, and dot-probe methods to study attentional bias to food-related stimuli [23]. Of note, no previous study has taken stimulus novelty and familiarity into consideration, nor how the results of this integrated approach associate with the self-report food choices. Familiar stimuli evoke trust and perceived quality from previous positive experiences are suggested to influence an individual’s choices [24]. However, novel products require more cognitive effort to evaluate, leading to longer information processing times [25]. Thus, the main objective of this pilot study is to examine differences in attention to novel compared with familiar food stimuli, using 3 different attention indexes, including electroencephalography, eye tracking, and dot-probe. Indeed, it is hypothesized that participants will exhibit shorter reaction times (RTs) and longer total fixation durations (TFDs) for familiar compared to novel food items, indicating a stronger attentional bias toward familiar foods. In addition, familiar food items will elicit greater P300 amplitudes compared with novel food items, suggesting enhanced attentional engagement and processing.

## Methods

### Objectives

The secondary objectives of this study are to obtain preliminary data on the quality of the data, along with the potential correlations between visual attention metrics as assessed by eye tracking, RTs in response to the dot-probe task, and amplitude of ERP components. In addition, an exploratory objective of this study is to investigate the association between the data of attentional bias tools with the self-reported food choice to further our understanding of the potential of ERP and eye-tracking data in predicting the individual’s ultimate food choice, particularly taking stimulus familiarity into consideration.

### Study Design

This nonrandomized pilot study aims to use different measures of attention, via an integrated eye tracking and electroencephalography methodology, to study different aspects of the food-related attentional bias. Synchronization will ensure that eye movements and neural responses are recorded concurrently in response to the same stimuli during the dot-probe task, allowing precise alignment of data for accurate analysis.

### Study Setting

Data collection will be completed at the Iran University of Medical Sciences in Tehran, Iran, by trained research assistants and technicians.

#### Eligibility Criteria

The inclusion criteria include participants (1) aged between 18 and 50 years; (2) with a BMI within the healthy range (18.5-24.9 kg/m^2^), to avoid confounding effects of weight status on food preferences and attentional biases; (3) with normal or corrected-to-normal vision (required for accurate eye-tracking data and visual task completion; (4) who are right-handed, to control for hemispheric dominance effects in electroencephalography recordings, as handedness can influence attentional processing and ERP components (eg, P300, N100), reducing variability in neural responses; (5) with no history of neurological or psychiatric disorders, to minimize potential confounding factors that could influence the study outcomes; (6) with no use of medications affecting cognitive function or appetite including psychiatric medications, stimulants, or appetite suppressants; and (7) who are willing to complete the study tasks, including questionnaires, electroencephalography recordings, eye-tracking, and the dot-probe task.

The exclusion criteria include individuals with (1) a history of eating disorders or current disordered eating patterns; (2) a history of visual or motor impairments; (3) high sensitivity to visual stimuli, including photosensitivity, severe migraines, and epilepsy; or (4) an unwillingness to adhere to study protocol or incomplete or insufficient data collection during the study.

#### Visual Stimuli Selection

We aim to identify the perceived familiarity of a range of food items among a test sample to categorize them into “highly familiar,” “familiar,” “somewhat familiar,” or “novel” for the main study. This will ensure that the familiar or novel food stimuli in the main experiment reflect participants’ everyday experiences. In this regard, we will create a list of food items, including a diverse range of food items from the categories we plan to use in the main study (eg, milk, oil, sauces, and beverages). Food products of “everyday” life, which have moderate visual complexity (no complex dishes), will be chosen as possible stimuli. To achieve a balanced set, we will include items commonly found in the target population’s diet (eg, whole milk, olive oil) and novel items they are less likely to encounter (eg, hemp milk, truffle oil). We will organize the food items into the categories we will be using in the main study to facilitate easy comparison (eg, “Beverages” might include orange juice, kombucha, and matcha latte). Accordingly, we will create a structured survey using Google Forms where each item will be rated based on familiarity using a Likert scale: 1=highly unfamiliar (I have never seen or heard of this product); 2=somewhat unfamiliar (I have rarely seen or heard of this product); 3=somewhat familiar (I know of this product but don’t use it often); 4=familiar (I use this product occasionally); and 5=highly familiar (I use this product regularly). We aim to recruit 100 participants to ensure diverse responses, taking demographic diversity (eg, age, gender, dietary preferences) into account to get a representative sample. Before launching the main survey, we will conduct a small pilot test with a handful of individuals (5-10 people) to identify any issues with wording, scale clarity, or question order.

Finally, we will calculate the mean familiarity score for each food item and classify items based on their mean familiarity score, based on the following scores:

- Highly familiar: average score of 4-5
- Familiar: average score of 3-4
- Somewhat familiar: average score of 2-3
- Unfamiliar: average score of 1-2

Items falling into the “Highly Familiar” or “Familiar” categories will be used as familiar stimuli, while those in the “Unfamiliar” category can serve as novel stimuli for the main study. Based on the survey results, we will select the top 2 items from each category (eg, milk, oil, sauces, biscuits, and beverages) to be used as stimuli in the main study. Special care will be taken to select pictures of good quality, comparable lighting, and size. The pictures of each pair will be shown side by side and matched as closely as possible with regard to the shape, color, and position of the photographed object as well as background color. One choice out of the 5 choice sets (milk, oil, sauces, biscuits, and beverages) will be used as a warm-up to familiarize the participants with the procedure and will not be included in the data analysis.

#### Intervention

Participants who meet the inclusion criteria will be recruited to complete a dot-probe task while integrated electroencephalography and eye-tracking data are recorded. Each participant will conduct a series of attention-related tasks (an eye-tracking task, a visual probe task, and an ERP task), and, hereafter, a Food Choice Questionnaire (FCQ) to assess food choice. The intervention involves presenting participants with both familiar or novel food items from 4 categories (milk, oil, sauce, and beverages) simultaneously during the dot-probe task to assess attentional biases. In this task, pairs of images (one familiar and one novel food item) will be displayed on a computer screen, followed by a dot-probe appearing in the location of either the familiar or novel item. Participants will be instructed to respond as quickly and accurately as possible by pressing a key corresponding to the location of the dot-probe. Reaction times to the dot-probe will be recorded. Neural responses will be captured using electroencephalography to identify specific brain activity patterns associated with processing the food stimuli, offering insight into the cognitive and neural mechanisms underlying attention. Eye-tracking technology will also be used concurrently to monitor gaze patterns and fixation duration (FD) on each food item, enabling us to quantify the amount of visual attention allocated to familiar versus novel items. No intervention modifications are planned in this study, as they do not apply to the type of intervention used.

#### Procedure

On the day of the main study, participants will first complete a demographic questionnaire. After this, they will have a brief period to relax and prepare for the tasks ahead. The study will use 3 attention indexes, all featuring the same food item images: an eye-tracking paradigm (measuring gaze direction and duration), a visual dot-probe task (assessing reaction times), and the recording of electrophysiological brain activity (evaluating the amplitude of the P300 and N100 ERPs). Before starting the tasks, electrodes will be positioned and adjusted, using the Cz electrode as the reference point (located at the center of the electroencephalography cap). A potassium chloride solution will be applied to the electrodes’ sponges to lower impedance to below 5 kΩ. Participants will then be seated 60 cm away from the eye-tracking system, with the camera calibrated to optimally capture their eye movements, followed by a 9-point calibration procedure to ensure accurate eye-tracking data. After successful calibration, participants will be instructed to keep their heads still and focus on the images displayed on the screen. They will read the instructions and complete 5 practice trials to familiarize themselves with the task.

Briefly, participants will be instructed to respond as quickly and accurately as possible by pressing a key corresponding to the dot’s location during a dot-probe task, with ERPs being recorded via electroencephalography. Participants’ gaze position will also be tracked every 16 milliseconds (60 Hz) throughout the experiment. The entire experiment will take place in a controlled environment, minimizing electromagnetic interference and maintaining consistent lighting. Once the integrated methods are completed and all recordings have been captured, participants will be asked to fill out the food choice questionnaire.

### Outcomes

#### Primary Outcome

The primary outcome includes identifying the difference in 3 attentional bias metrics toward familiar or novel food stimuli at different presentation times. Detailed descriptions of these outcome measures are provided in the “Outcome Measures” section below.

#### Secondary Outcomes

The secondary outcomes focus on evaluating the correlations between ERP data obtained from electroencephalography recordings and the metrics captured through eye tracking and RTs from the dot-probe task.

#### Exploratory Outcomes

Exploratory outcomes investigate the effects of food familiarity or novelty on food choices and assess the potential of visual attentional bias data, derived from 2 well-established tools, to predict final food choices among healthy adults.

### Outcome Measures

In this study, several key metrics will be used to assess visual and neural responses to food stimuli, which are listed in Textbox 1.

**Outcome measures.**

**Time to first fixation (TTFF)**

The interval from the onset of the stimulus to the first fixation on the target. TFF indicates the initial orienting response towards a stimulus. A shorter TTFF suggests quicker attentional capture by the target, reflecting the efficiency of visual attention and its alignment with task-relevant stimuli [26]. This measure is crucial for understanding how quickly participants direct their gaze to familiar versus novel stimuli.

**Total fixation duration (TFD)**

The cumulative time spent fixating on a target during a given trial. TFD reflects the allocation and engagement of attention over time. A longer TFD on a target implies greater sustained attention and interest, indicating how engaging or relevant the stimulus is perceived to be. This measure elucidates how familiarity affects sustained visual attention toward different products [27].

**Reaction times (RTs)**

The time taken by participants to respond to the dot-probe after it appears, measured from the onset of the probe. RTs provide insights into attentional biases and decision-making speeds. Shorter reaction times suggest faster processing and quicker attentional shifts towards the location of the dot-probe, helping determine how stimulus familiarity influences the speed of attention allocation and the efficiency of task-related responses [15].

**Event-related potential (ERP) components**

ERP components capture the neural responses associated with processing visual stimuli. A total of 2 key components will be analyzed:

- P300: A positive deflection occurs approximately 300-600 milliseconds post stimulus. The P300 component is linked to attention and memory processes, reflecting the allocation of attentional resources and the processing of significant or novel stimuli. Higher P300 amplitudes suggest greater attentional engagement and relevance of the stimulus, providing insights into how product familiarity influences cognitive processing [28].
- N100: A negative deflection occurs approximately 100 milliseconds post stimulus. The N100 component is associated with early sensory processing and selective attention, indicating the initial detection of and orienting toward a stimulus. Variations in N100 amplitude can reveal differences in early attentional processing between familiar and unfamiliar products [29].

### Participant Timeline

The protocol was prepared according to the SPIRIT (Standard Protocol Items: Recommendations for Interventional Trials) guidelines (the SPIRIT checklist can be found in Multimedia Appendix 1) [30]. In addition, Table 1 provides an overview of the study timeline.

**Table 1:** SPIRIT (Standard Protocol Items: Recommendations for Interventional Trials) report of enrollment, interventions, and assessments.

Study period		Enrolment (–t_1_)	Allocation (0)	Postallocation (t_1_)	Close-out (t_2_)
**Enrolment**					
	Eligibility screen				
	Informed consent	✓			
	Demographic data	✓			
**Interventions**					
	Allocation		✓		
	Dot-probe task			✓	
**Assessments**					
	Reaction times			✓	
	Eye-tracking metrics			✓	
	Event-related protentional components (P300, N100)			✓	
	Food choice questionnaire				✓

### Sample Size

An a priori power analysis was conducted using G Power version 3.1.9.7 (Department of Psychology, Heinrich Heine University Düsseldorf) to determine the appropriate sample size needed to achieve sufficient statistical power for our study. The analysis was designed for an *F* test, with an assumed medium effect size of *F*=0.35, a significance level of α=.05, and a desired statistical power of 0.80. Based on these parameters, the calculated sample size required was 40 participants. Specifically, the number of groups (between-subject factor) was set at 2 (500 ms vs 2000 ms), and the number of measurements (within-subject factor) included 8 (2 stimulus types [familiar or novel] across 4 food categories). In addition, the correlation among repeated measures was conservatively set at 0.5. The medium effect size was chosen based on the existing literature on attentional bias studies, which commonly report medium to large effect sizes [23]. Under these specifications, G Power estimated an achieved power of 0.820, exceeding the commonly accepted threshold of 0.80 for adequate power. This indicates that a sample size of 40 participants is sufficient to detect a medium effect size in our primary outcome using repeated measures ANOVA, ensuring the study is powered to identify meaningful changes while balancing feasibility within the constraints of a pilot study.

### Recruitment

Participant recruitment will be conducted using a multifaceted approach, combining both physical and internet-based strategies. This will include distributing flyers in community centers, libraries, and university campuses, as well as posting digital flyers on social media platforms (eg, Instagram). We will also use email invitations, university newsletters, and internet-based research recruitment platforms. Potentially eligible individuals will be identified, and members of the study team will reach out to them via phone or email to provide detailed information about the study and extend an invitation to participate. Interested individuals will be directed to complete an electronic screening survey to assess eligibility. Those who qualify and express interest will be contacted by the study team to verify screening information. During this meeting, the study team will review the study procedures, answer any questions, and go over the consent process.

### Data Collection Plan

#### Dot-Probe Task

**Figure 1:**
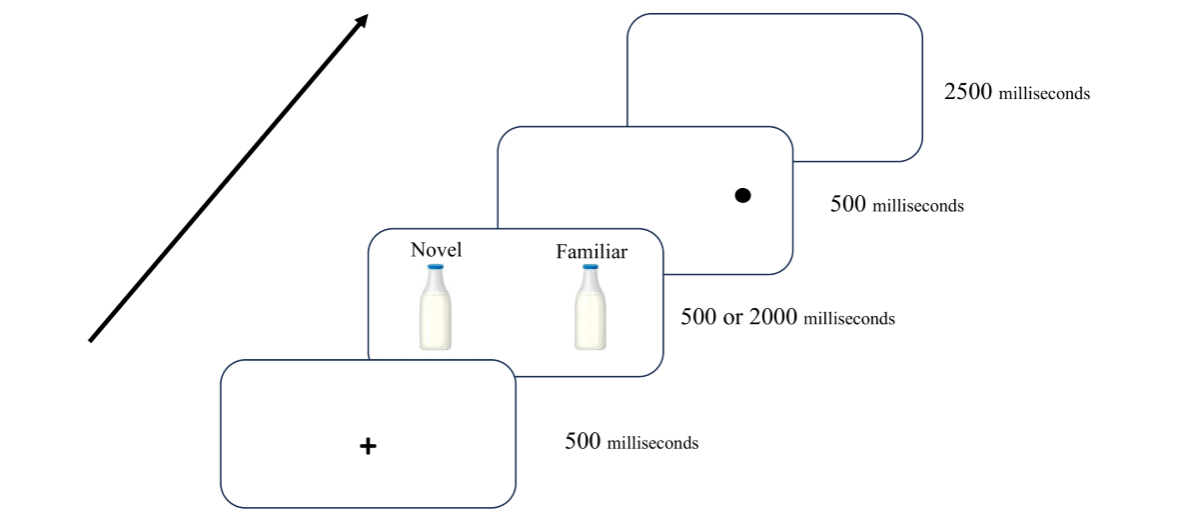
Schematic view of the dot-probe task design.

The dot-probe task will be implemented using E-Prime 3.0 software (Psychology Software Tools, Inc) for both eye-tracking and ERP components. Each trial will commence with a 500-millisecond presentation of a fixation cross at the center of the screen. This serves to focus participants’ attention on the central point before the food stimuli are displayed. Following the fixation cross, a pair of images (one familiar and one novel) will be shown side-by-side for either 500 or 2000 milliseconds. The side on which each type of image appears is randomized to control for location bias. After the image pair is presented, a dot-probe will appear in the location previously occupied by one of the images. The dot will remain visible for 500 milliseconds. Participants will indicate the location of the dot by pressing the “L” key if the dot appears on the left side of the screen, or the “R” key if it appears on the right side. The response time and accuracy will be recorded for each trial (Figure 1).

Each image pair will be displayed 8 times throughout the experiment, ensuring all possible dot-probe locations are tested. This results in a total of 128 trials, with varying combinations of familiar or novel images appearing in each trial. Reaction times in all trials will be recorded. RTs refer to the time it takes for participants to respond to the target stimulus by pushing the right or left key after the presentation of the stimulus. RTs are extracted as a measure of the speed at which individuals can shift their attention from the cue to the target. The inter-trial interval will be 2500 milliseconds. Reaction times of incorrect responses as well as reaction time outliers (reaction times less than 200 ms, greater than 1500 ms) will be excluded from the analyses. Temporal dynamics refer to variations in the duration of stimulus presentation. Shorter durations (eg, 500 ms) are often used to capture initial orienting responses. A quick reaction during a brief presentation may indicate a rapid attentional capture by the cue. Longer durations (eg, 2000 ms) are used to explore sustained attention and processing.

#### Eye Tracking

The eye-tracking component of this study will be conducted using the Tobii Nano Pro (Tobii Tech), a screen-based eye tracker known for its high precision in capturing gaze data. The Tobii Nano Pro offers a sampling rate of 60 hertz, which translates to data collection at intervals of 16.67 milliseconds. This high frequency allows for detailed tracking of eye movements and gaze patterns. To ensure accurate gaze data, a standard 9-point calibration procedure will be performed at the beginning of each session. This process involves directing the participant’s gaze to 9 specific points on the screen to map their eye movements accurately. The calibration will be verified for precision before starting the main trials to ensure reliable spatial and temporal tracking. Participants will be seated approximately 60 cm from the Tobii monitor, which has a resolution of 1600 × 1200 pixels. Participants will be instructed to maintain a still head position and focus attentively on the images displayed on the screen. Participants will view pairs of pictures displayed side by side for 500 or 2000 milliseconds each. Each image pair is matched for shape, color, and background to control for these variables. The following eye-tracking parameters will be measured:

1. TTFF: time elapsed between the appearance of a picture and the user’s initial gaze fixation within an area of interest. In this case, each product will be defined as a separate area of interest.
2. First fixation duration: time participants gaze at their first fixation point.
3. TFD: length of all fixations within a specific area of interest (in seconds). Average TFD will be used for statistical analyses.
4. Fixation count: number of fixations on stimuli.
5. Eye movement data will be analyzed using Tobi software.

Fixations are defined as periods where the gaze remains stable within a 30-pixel radius for at least 100 milliseconds, and these fixations must be initiated at least 100 milliseconds after picture onset to exclude anticipatory eye movements.

#### Electroencephalography Acquisition

As an electrophysiological measure of attention, electroencephalography will be recorded during the exposure to food stimulus pictures to determine the P300 and N100 ERPs. Electroencephalography signals will be recorded using the 9-channel B-Alert EEG system, which captures neural signals at a sampling rate of 256 hertz, providing data acquisition approximately every 3.91 milliseconds. This setup ensures the high temporal resolution necessary for detailed analysis of ERPs. Electrodes will be positioned following the international 10-20 system to ensure consistent and reliable data collection. The 9-channel configuration will cover standard locations to accurately capture neural signals. To maintain a clear signal acquisition, the impedance for each electrode will be kept below 5 kΩ, and impedance levels will be checked before data collection begins. An additional 2 electrodes will serve as reference and ground electrodes to improve the accuracy of the recordings. To address ocular artifacts, additional electrodes will be placed at the left and right mastoids, as well as at the supraorbital and suborbital positions of the left eye (for vertical electrooculography, VEOG), and the outer canthi of both eyes (for horizontal electrooculography, HEOG). Then, preprocessing will be performed using the B-Alert software (Advanced Brain Monitoring, Inc) to remove noisy segments of the signal. Subsequently, the signals will be epoch-referenced, with epochs spanning from the onset of the dot presentation to 1000 milliseconds after the presentation of the stimuli. The signal will be filtered at 50 hertz by the device, so there will be no need for additional filtering for power line noise and harmonics. Under appropriate conditions, the amplitude of the P300 indexes response categorization and the depth of encoding of the stimuli; larger amplitude P300s are thought to represent the amount of processing necessary to update neural networks about the changing environment. While there are a number of complementary and competing models of P300 [28], and several identified factors that affect the amplitude and latency of the P300 [31], all agree that stimuli garnering more attentional resources produce larger amplitude P300s. The collected electroencephalography data will be preprocessed using the EEGLAB environment, a tool library for processing electroencephalography data in MATLAB. The adjust plugin in EEGLAB will be used to discard artifacts due to eye movement and muscle activity.

#### Software Coordination

The E-Prime software will be integral for stimulus presentation and task control. It will manage the timing of stimulus presentation and coordinate trigger signals to synchronize the eye-tracking and electroencephalography systems. E-Prime will insert data markers into both the eye-tracking and ERP data streams at the beginning and end of each stimulus presentation, ensuring precise alignment of the data during analysis. For temporal accuracy, eye-tracking data will be aligned with ERP data using timestamps from the synchronization triggers, ensuring that each data point from the eye-tracker corresponds to the same point in time as the neural data. Any potential latency differences between the systems will be identified and corrected during the data analysis phase using these synchronized timestamps. A custom script in MATLAB will integrate the eye-tracking and ERP data based on synchronization markers provided by E-Prime.

### Questionnaires

#### Demographic

The demographic questionnaire will be used to collect baseline information on participants to ensure that the study population is adequately characterized. The demographic questionnaire will collect information on age, gender, education level, socioeconomic status, smoking status, etc. The data gathered will be used for descriptive statistics, to stratify analyses, and to ensure that participants meet eligibility criteria.

#### Food Choice Questionnaire

Participants will be asked to self-report their final selection from the food items presented during the study. They will also be asked to complete a 36-item FCQ [32] for each familiar or novel item in each food group. FCQ consists of 9 subscales designed to assess the significance of various factors influencing food choices. These subscales measure the importance of health considerations, convenience in terms of preparation and purchase, price and value for money, sensory appeal (including taste, smell, and texture), natural content and the absence of additives, mood regulation, and the use of food to manage stress, familiarity with the food, ethical concerns related to origin and packaging, and weight control (the questionnaire can be found in Multimedia Appendix 2).

#### Data Management

All data collected during the study will be securely stored and managed to ensure participant confidentiality and data integrity. Each participant’s data will be assigned a unique identifier to maintain anonymity. Data from the dot-probe task, eye-tracking, and electroencephalography recordings will be directly entered into secure, password-protected databases. Data entry will be verified by cross-checking with the original source files to ensure accuracy. All electronic data will be stored on encrypted servers with regular backups. Physical data, such as consent forms, will be stored in locked cabinets accessible only to authorized personnel. Access to the data will be restricted to the research team members directly involved in the study. Any data sharing with external collaborators will be conducted under strict data-sharing agreements ensuring confidentiality and compliance with ethical standards.

### Statistical Analysis

ERP data will be preprocessed with EEGLAB v14.1.2 toolbox for MATLAB [33], involving noise removal, epoching from stimulus onset to 1000 milliseconds post stimulus, and averaging across epochs to extract ERP components. Potentials reflecting ocular or muscle-related artifacts were removed using logistic infomax independent component analysis [34]. Further artifacts will be removed using the clean_rawdata plugin for EEGLAB.

Regarding dot-probe and electroencephalography attention-related measures (RTs, and N100 and P300 amplitudes, respectively), repeated measures ANOVA will be conducted, including the stimulus type (familiar vs novel across 4 food categories) as a within-subject factor, and group (500 ms vs 2000 ms) as a between-subject factor. Similarly, ANOVA will also be conducted to examine eye-tracking measures (TTFF, FD) between groups (500 ms vs 2000 ms). Post-hoc analyses will be conducted with Bonferroni adjustments for multiple comparisons. In addition, relationships between eye-tracking measures (TTFF, FD) and ERP components (P300 and N100 amplitudes) will be calculated by the Spearman correlation analysis. IBM SPSS Statistics 20 (IBM Corp) software will be used to analyze the data. No interim analyses are scheduled for this study.

### Ethical Considerations

The study protocol has been approved by the Research Ethics Committee of Iran University of Medical Sciences (ethics approval code: IR.IUMS.REC.1399.1340), in accordance with institutional and national ethical standards and the principles outlined in the Declaration of Helsinki.

Before consent, prospective participants will be sent a copy of the consent form to review and discuss further with family and friends if needed. Potential participants will then attend a pre-enrollment consent review call (phone or internet-based). Participants will be informed that participation is voluntary and that they may leave the study at any time without prejudice. When the study participant arrives at the Brain and Cognition Clinic, Iran University of Medical Sciences, any additional questions will be answered, and additional explanations will be provided as needed before they will be asked to sign and date their agreement to participate in the study. A paper or scanned copy of the signed consent form will be provided to the study participant for their records.

All participant data will be anonymized to ensure no individual is identifiable in any published report or supplementary materials. No images or information that could identify individual participants will be included. Each member of the research team has been trained in maintaining confidentiality to ensure the protection of participant information throughout the study.

Participants will receive monetary compensation upon completion of the experiment to acknowledge their time and effort. The compensation (US $15) will be provided in the form of a gift card and does not require any financial contribution from participants.

## Results

This study was approved by the Ethics Committee of the Iran University of Medical Sciences in February 2021 and funded in January 2022. Data collection officially commenced in November 2022 and is projected to conclude by July 2025. As of the time of this study’s submission, 34 participants have been successfully enrolled in the study, aligning with recruitment goals. Data analysis is planned to start immediately following the completion of data collection to ensure a timely and thorough evaluation of the findings. The study results are expected to be finalized and submitted for publication by December 2025. In addition, this protocol was registered with the Open Science Framework in September 2024 (osf.io/ctfyb), emphasizing the study’s commitment to transparency and open science practices.

## Discussion

### Principal Findings

This study aims to investigate the attentional biases associated with food stimuli, comparing familiar or novel food items, using different attention indexes. We hypothesize that participants will exhibit shorter RTs and longer TFD for familiar food items compared with novel food items, suggesting a stronger attentional bias toward familiar foods. In addition, we expect familiar food items to elicit greater P300 amplitudes in electroencephalography recordings, indicating enhanced attentional engagement and processing. Furthermore, we anticipate that eye-tracking measures will correlate with electroencephalography-derived ERP components, highlighting the joint contribution of neural and ocular markers to attentional bias toward food stimuli. Finally, we anticipate that various attentional bias measures, including RTs, ERP amplitudes, and eye-tracking metrics, would predict self-reported food choices, demonstrating the relevance of attentional mechanisms in consumer behavior.

While previous research has explored the effects of familiarity on consumer preferences and decision-making [35,36], the combined use of eye-tracking and electroencephalography metrics remains underexplored, particularly when taking the participant’s food choice into consideration. The integration of these technologies allows for a comprehensive analysis of both spatial and temporal aspects of attention, capturing where and how long participants focus on food stimuli and the neural dynamics underlying these attentional processes [37]. Previous research has highlighted the relevance of visual attention in the food decision-making process. For instance, in the study by Gere et al [38], the authors proposed using alternative models to analyze visual attention patterns during food choices. Their approach emphasized the use of multimodal data, such as eye-tracking and neurophysiological measures, to predict food preferences and decision outcomes more accurately, which supports the use of combined methodologies in our pilot study to capture these intricate cognitive processes. In addition, Wang et al [39] used eye-tracking and electroencephalography to investigate how emotional states influence attention to food-related stimuli. The findings revealed distinct neural patterns associated with processing positive versus negative stimuli and suggested that emotional context significantly modulates visual attention towards food. This insight aligns with our aim to explore potential neural mechanisms (eg, P300 and N100 ERP components) that may underlie attentional biases in response to food stimuli, indicating that factors such as familiarity and novelty could similarly impact cognitive responses. Furthermore, research comparing attentional differences in overweight or obese versus normal-weight females under hunger and satiety conditions demonstrated that hunger states amplify attention to high-calorie foods, while satiety reduces such biases [23]. These findings suggest that attentional biases are dynamic and can be influenced by both physiological states and personal food-related experiences.

Integrating these perspectives, our study seeks to build upon these observations by investigating how familiarity and novelty interact with attentional biases, potentially offering a new understanding of how these factors shape food choices. A recent study investigating electroencephalography measures of attention toward food-related stimuli in neophobic individuals proposed that food neophobia might be associated with attentional biases toward novel foods. However, the results indicated that implicit behavioral and attentional processing were affected across all food types, regardless of familiarity [40]. This finding, alongside several other studies [23,41] examining attentional differences toward food stimuli based on factors such as calorie content and familiarity across diverse populations, underscores the importance of using multiple methods to capture different dimensions of attention. This study serves as an initial step in piloting an integrated approach, combining electroencephalography, eye tracking, and a dot-probe task, to gather preliminary data on the feasibility of using this multimodal framework to assess attentional responses to familiar versus unfamiliar food stimuli in healthy adults. A recent systematic review and meta-analysis on attentional bias toward food in individuals with overweight and obesity [42] highlighted the poor reliability of eye tracking and the dot-probe task, 2 commonly used methods for measuring attentional bias. The review emphasized that the poor psychometric properties of these measures represent a significant limitation in attentional bias research. In light of this, the present study’s integration of 3 different attentional bias tools is a key strength, offering a more comprehensive approach to capturing multiple dimensions of attentional processes. In addition, this study has several other strengths. First, the integration of eye-tracking, electroencephalography, and the dot-probe task is a novel approach that allows for a comprehensive analysis of attentional biases towards food stimuli. Second, by examining attentional biases toward familiar or novel food stimuli, the study addresses an important aspect of consumer behavior with implications for both health behaviors and marketing strategies. Third, the use of multiple measures (eg, TTFF, TFD, RTs, P300, N100) enhances the reliability of the findings and provides a solid foundation for validating results in future studies.

However, the study protocol is also subject to several limitations. The relatively small sample size of 40 participants, while typical for pilot studies, may limit the generalizability of the findings and reduce statistical power to detect smaller effects. In addition, the lack of a more diverse sample, including individuals who are overweight or obese, a wider age range, and not including the satiety status, may limit the generalizability of the findings to broader populations. The simultaneous use of electroencephalography and eye-tracking introduces potential technical challenges and artifacts in the data, which could affect measurement accuracy. Finally, the study’s focus on a limited number of food categories (milk, oil, sauces, and beverages) may not fully capture the range of food stimuli that influence attentional biases and food choices.

Following the pilot study, conducting larger-scale studies with more diverse samples would enhance the generalizability and robustness of the findings. This could involve multiple sites and a broader range of food categories.

### Dissemination Plans

The results will be submitted to peer-reviewed journals in fields such as neuroscience, psychology, nutrition, and consumer behavior. This ensures that the findings are scrutinized by experts and accessible to the academic community. Presenting the findings at national and international conferences will facilitate knowledge exchange with other researchers and practitioners. Organizing workshops at universities and research institutions to share the methodology and findings with students and faculty. This can also include training sessions on the integrated use of eye-tracking and electroencephalography. Collaborating with policy makers, health organizations, and the food industry to translate the research findings into practical strategies for promoting healthier eating habits and informing marketing practices.

In summary, the integration of eye-tracking and electroencephalography data in our study will allow for a detailed examination of attentional processes, building on previous research that underscores the importance of capturing both visual and neural indicators when exploring food-related decision-making. Such insights will not only enhance our understanding of attentional mechanisms in food choice but also contribute to developing strategies to promote healthier food decisions.

### Conclusion

Future directions could include developing and testing interventions based on the findings, such as strategies to modify attentional biases toward healthier food choices, which could have practical applications in public health and consumer behavior. Notably, expanding the research to include different populations, such as individuals with obesity, eating disorders, or diverse cultural backgrounds, would provide a more comprehensive understanding of attentional biases in various contexts and further inform intervention strategies.

## Appendix

Multimedia Appendix 1 SPIRIT (Standard Protocol Items: Recommendations for Interventional Trials) checklist.

Multimedia Appendix 2 Food Choice Questionnaire.

## Abbreviations

ERP: event-related potential
FCQ: Food Choice Questionnaire
FD: fixation duration
RT: reaction time
SPIRIT: Standard Protocol Items: Recommendations for Interventional Trial
TFD: total fixation duration
TTFF: time to first fixation

## Data Availability

Given the nature of this study protocol, no data or materials are currently available. Upon study completion, all data generated or analyzed will be included in the published article and its supplementary information files.
