# Supplementary material for: Exploring the Link Between Visual Attention to Familiar or Novel Food Stimuli and Food Choice Using Integrated Electroencephalography and Eye Tracking: Protocol for Nonrandomized Pilot Study
Source: JMIR Res Protoc. 2025 May 21;14:e69541. doi: 10.2196/69541 (PMC12138311; doi:10.2196/69541)
Supplement: Multimedia Appendix 2 [file resprot_v14i1e69541_app2.pdf]

Food Choice Questionnaire

Subscales	Items
Health	Is high in fibre and roughage<br>Is nutritious<br>Contains lots of vitamins and minerals<br>Is high in protein<br>Keeps me healthy<br>Is good for my skin/teeth/hair/nails etc.
Mood	Cheers me up<br>Helps me cope with stress<br>Keeps me awake and alert<br>Helps me relax<br>Makes me feel good
Convenience	Is easily available in shops and supermarkets<br>Can be cooked very simply<br>Takes no time to prepare<br>Can be bought in shops close to where I live or work
Sensory appeal	Tastes good<br>Smells nice<br>Looks nice
Natural content	Contains no additives<br>Contains natural ingredients<br>Contains no artificial ingredients
Price	Is not expensive<br>Is good value for money<br>Is cheap
Weight control	Is low in calories<br>Is low in fat
Familiarity	Is familiar to me<br>Is like the food I ate when I was a child<br>Is what I usually eat
Ethical concern	Is packaged in an environmentally friendly way<br>Comes from countries I approve of politically<br>Has the country of origin clearly marked.

My final choice in X food group is: …………

X: milk OR oil OR sauce OR beverages
